# Supplementary material for: The DYT6 dystonia causative protein THAP1 is responsible for proteasome activity via PSMB5 transcriptional regulation
Source: Nat Commun. 2025 Feb 14;16:1600. doi: 10.1038/s41467-025-56867-x (PMC11828994; doi:10.1038/s41467-025-56867-x)
Supplement: Supplementary file 1 — Supplementary Information [file 41467_2025_56867_MOESM1_ESM.pdf]

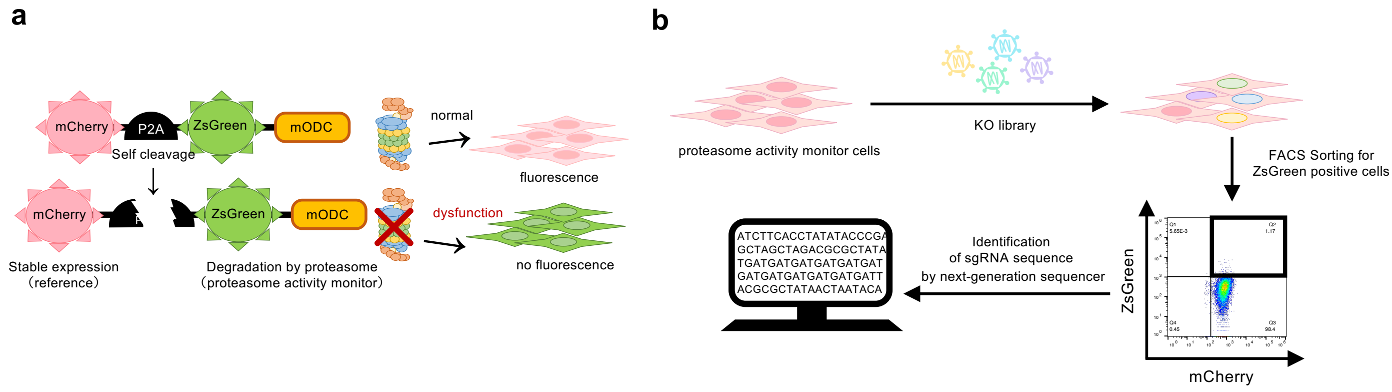

c

Top 50 genes

rank	genes	rank	genes
1	*PSMD11*	26	*PSMB5*
2	*PSMC6*	27	*TINF2*
3	*PSMC1*	28	*PSMA2*
4	*PSMD2*	29	*TOMM20L*
5	*PSMB7*	30	*PSMD8*
6	*PSMD3*	31	*RABGGTA*
7	*PSMD7*	32	*PSMA1*
8	*PSMA6*	33	*BRMS1L*
9	*PSMA4*	34	*PSMB3*
10	*PSMA5*	35	*DHRS1*
11	*PSMC4*	36	*THAP1*
12	*PSMB4*	37	*SNX6*
13	*PSMD14*	38	*ADAMTS9*
14	*PSMB2*	39	*LTB4R*
15	*PSMA3*	40	*MPP5*
16	*PSMC2*	41	*FITM1*
17	*PSMB1*	42	*EOGT*
18	*PSMC3*	43	*C14orf93*
19	*PSMA7*	44	*GMFB*
20	*PSMB6*	45	*POLE2*
21	*PSMD6*	46	*PNMA1*
22	*MGAT2*	47	*VCPKMT*
23	*PSMC5*	48	*C3orf38*
24	*PSMD12*	49	*TP53I13*
25	*PRMT5*	50	*TGM1*

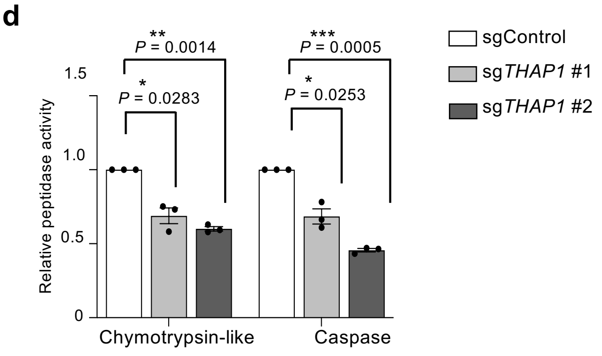

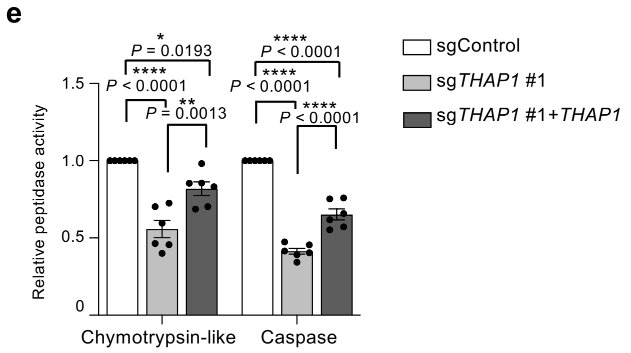

Supplementary Fig. 1 Genome-wide CRISPR knockout screening based on proteasome activity

a Schematic representation of the proteasome activity monitor. The fluorescent protein ZsGreen was fused to the mODC sequence (ZsGreen-mODC). mCherry, which serves as a reference, was linked with ZsGreen-mODC via the self-cleavage sequence P2A. **b** The schematic overview of genome-wide CRISPR knockout screening. The fluorescence intensity of ZsGreen relative to that of mCherry increases when proteasome function is impaired. The cell population in the upper right-hand quadrant of the flow cytometry plot was collected and subjected to next-generation sequencing analysis. **c** List of the top 50 hit genes identified in the screen. **d** Proteasome chymotrypsin-, trypsin-, and caspase-like activities of HEK293T cells transfected with sgControl or sg*THAP1*. The peptidase activities were measured in the presence of 0.025% SDS. **e** Proteasome chymotrypsin-like and caspase-like activities of the *THAP1* knockout cells in which sgRNA-resistant THAP1 cDNA was added back. The activities were measured in the presence of 0.025% SDS. Data represent the mean ± SEM (n = 3 from three biological replicates). The significance were calculated using an unpaired two-tailed Student's t-test with Welch's correction and one-way ANOVA Tukey with multiple comparisons test. *$P < 0.05$; **$P < 0.01$; ***$P < 0.001$; ****$P < 0.0001$. All experiments were performed at least three biologically independent times with similar results. Source data are provided as a Source Data file.

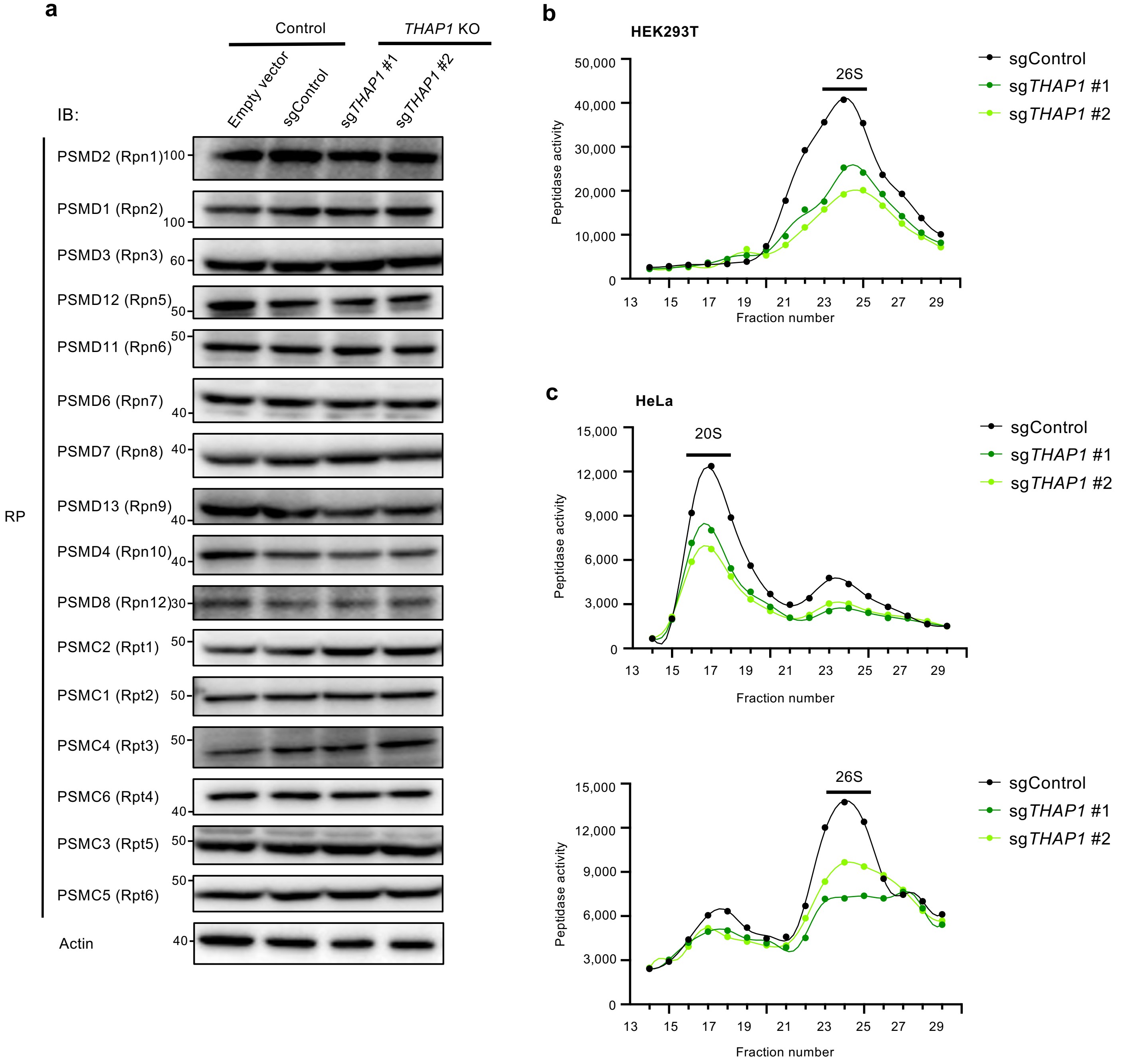

Supplementary Fig. 2 *THAP1* knockout does not affect RP subunits

a HEK293T cells lysate transfected with the indicated sgRNA was analyzed by immunoblot with antibodies against the indicated subunits. **b** The same samples shown in Fig. 2c were subjected to measurement of proteasome chymotrypsin-like activity in the absence of SDS. **c** Analyses analogous to those presented in Fig. 2c were performed using HeLa cells. An equal amount of each even-numbered fraction was used for measuring proteasome chymotrypsin-like activity in the presence (upper) or absence (lower) of 0.025% SDS. All experiments were performed at least three biologically independent times with similar results. Source data are provided as a Source Data file.

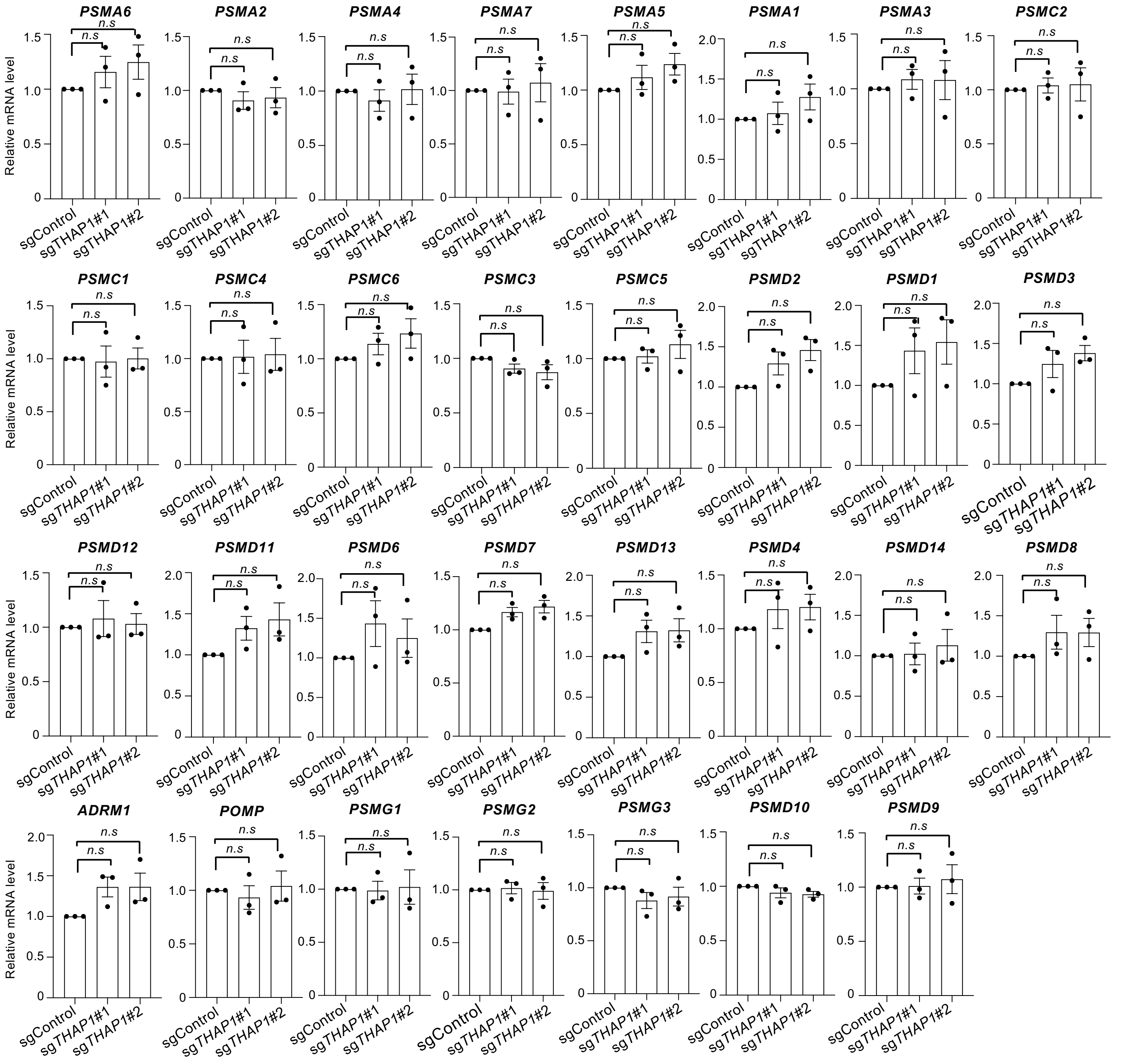

Supplementary Fig. 3 *THAP1* knockout does not affect the mRNA expression of α subunit and RP subunit genes

The mRNA expression analysis of the proteasome was conducted in parallel with the analysis presented in Fig. 3a. Data are presented as mean ± SEM (n = 3 from three biological replicates). The significance were calculated using an unpaired two-tailed Student's t-test with Welch's correction. *n.s., not significant.* All experiments were performed at least three biologically independent times with similar results. Source data are provided as a Source Data file.

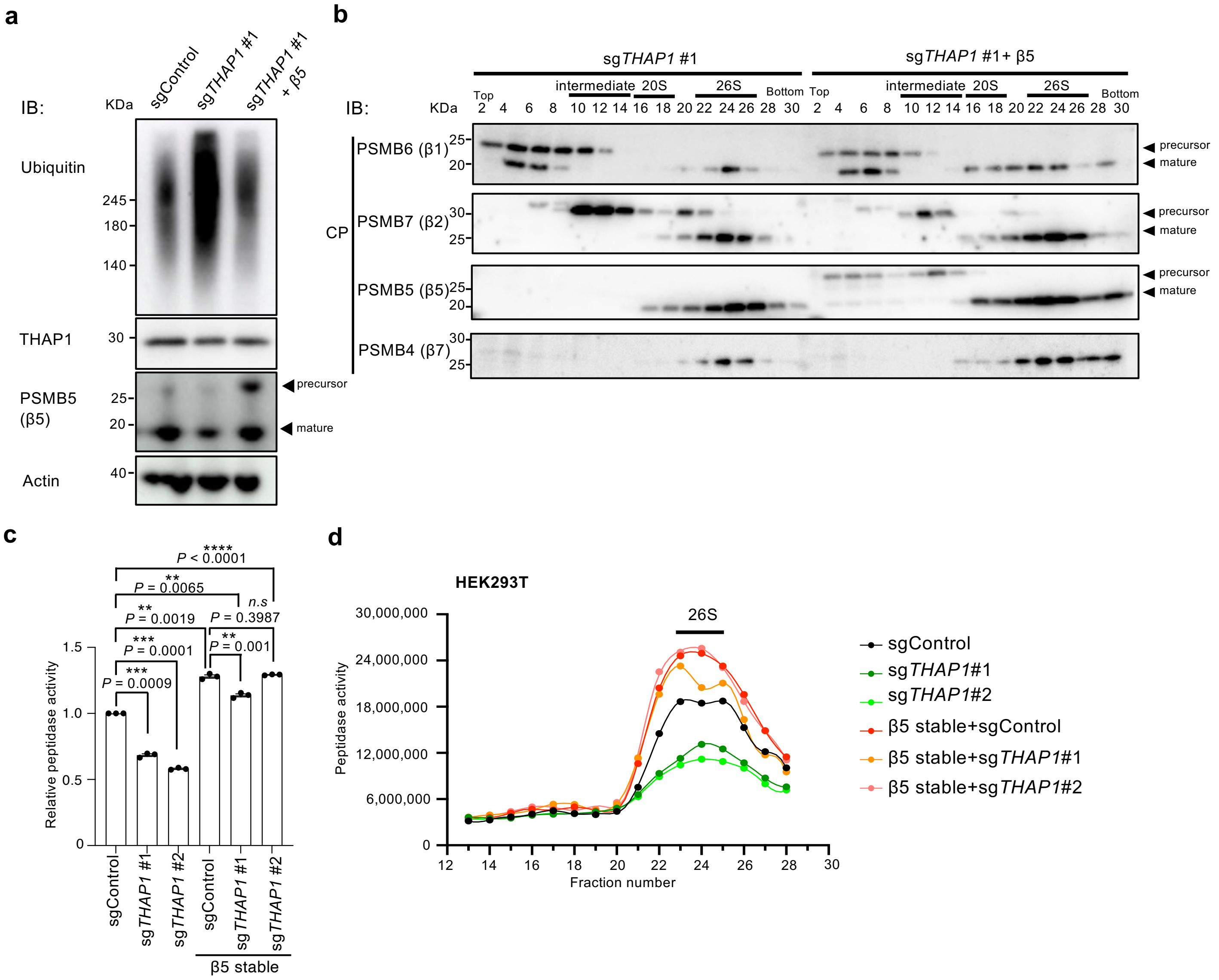

Supplementary Fig. 4 Overexpression of β5 restore the decreased proteasome activity caused by THAP1 knockout.
a The HEK293T cells were transfected with sgRNA targeting *THAP1* and subsequently transfected with each cDNA encoding proteasome β5 subunits. The cell lysates were subjected to immunoblot analysis using antibodies against the indicated proteins. **b** The lysates from (**a**) were fractionated by glycerol gradient centrifugation, and an equal amount of each even-numbered fraction was subjected to immunoblot analysis using antibodies against the indicated proteins. **c, d** Proteasome chymotrypsin-like activity assay (**c**) or with glycerol gradient fractionation (**d**) of HEK293T or β5-FLAG stably overexpressing HEK293T cells transfected with the indicated sgRNAs. Data are presented as mean ± SEM (n = 3 from three biological replicates). The significance were calculated using unpaired two-tailed Student's t-test with Welch's correction. *n.s., not significant*. $^{*}P < 0.05$; $^{**}P < 0.01$; $^{***}P < 0.001$; $^{****}P < 0.0001$. All experiments were performed at least three biologically independent times with similar results. Source data are provided as a Source Data file.

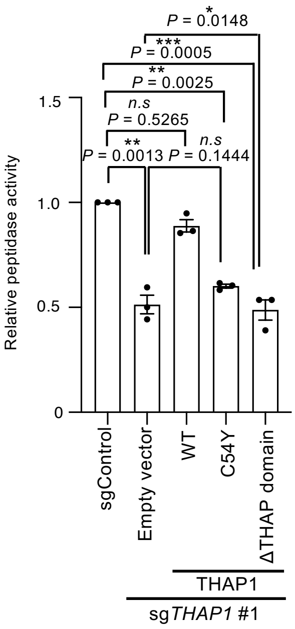

Supplementary Fig. 5 The THAP domain of THAP1 is essential for regulating proteasome activity.
Chymotrypsin-like activity of the *THAP1* knockout cells in which sgRNA-resistant cDNAs encoding wild-type THAP1, THAP1 C54Y, or THAP1 lacking THAP binding domain was added back. The activity was measured in the absence of SDS. Data are presented as mean ± SEM (n = 3 from three biological replicates). The significance were calculated using an unpaired two-tailed Student's t-test with Welch's correction. *n.s., not significant.* All experiments were performed at least three biologically independent times with similar results. Source data are provided as a Source Data file.

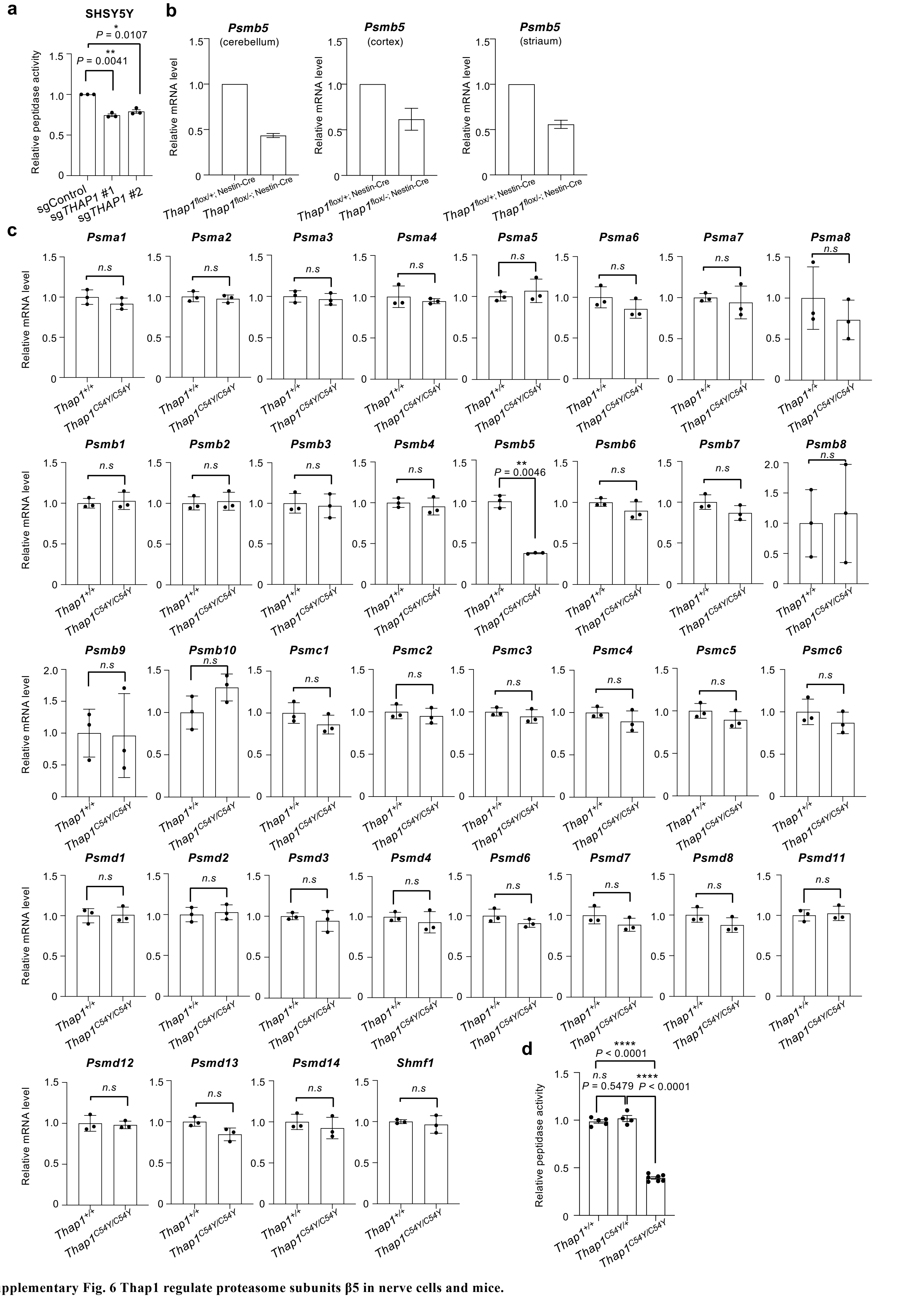

Supplementary Fig. 6 Thap1 regulate proteasome subunits β5 in nerve cells and mice.

a Proteasome chymotrypsin-like activity of SH-SY5Y cells transfected with sgControl and sg*THAP1*. The activity was measured in the absence of SDS. **b** *PSMB5* expression levels in the control (*Thap1*$^{flox/+; Nestin-Cre}$) and *Thap1* knockout (*Thap1*$^{flox/-; Nestin-Cre}$) cerebellum, cortex, and striatum were compared using microarray data from a previous study. **c** mRNA expression levels of all proteasome subunits in *Thap1* $^{C54Y/C54Y}$ embryos at E10.5. **d** Proteasome chymotrypsin-like activity of *Thap1* $^{C54Y/C54Y}$ embryos at E10.5. The activity was measured in the absence of SDS. Data are presented as mean ± SEM (n = 3 from three biological replicates). The significance were calculated using an unpaired two-tailed Student's t-test with Welch's correction and one-way ANOVA Tukey with multiple comparisons test. *n.s., not significant*. *P < 0.05; **P < 0.01; ***P < 0.001; ****P < 0.0001. All experiments were performed at least three biologically independent times with similar results. Source data are provided as a Source Data file.

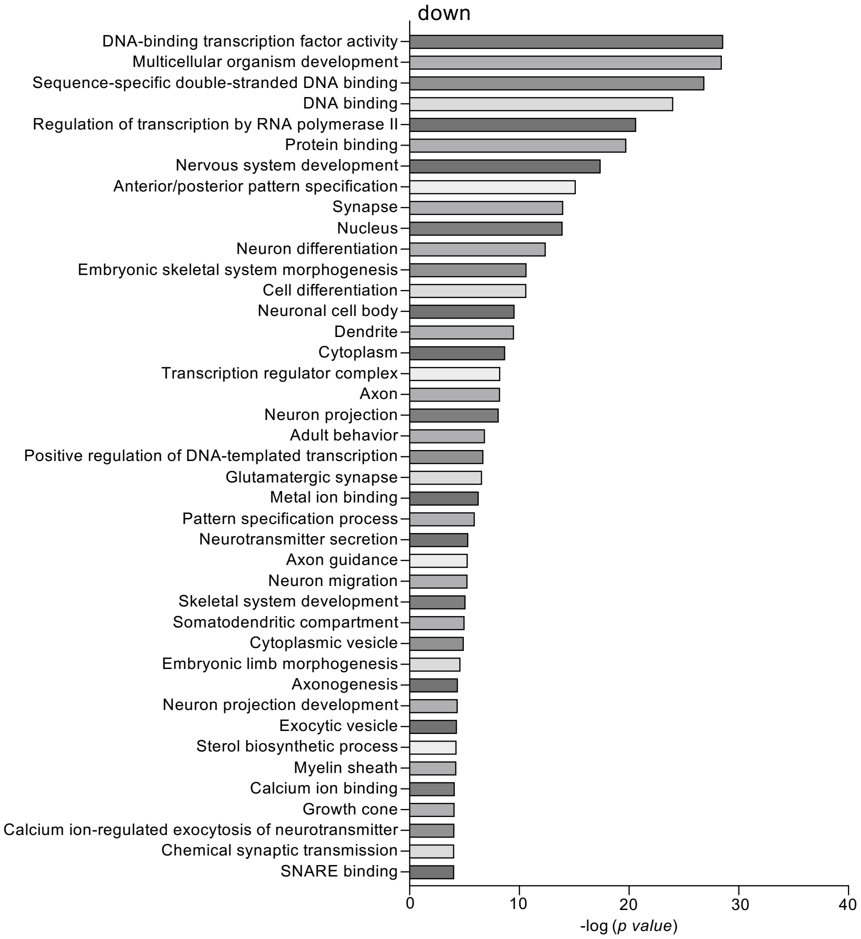

Supplementary Fig. 7 Development of *Thap1* $^{C54Y/C54Y}$ mouse embryos is impaired in the nervous system

Gene Ontology analysis of *Thap1*$^{C54Y/C54Y}$ embryos at E10.5, compared to control *Thap1*$^{+/+}$ embryos at E10.5. The significance were calculated using Modified Fisher Exact test and adjusted by Bonferroni, Benjamini, and FDR. Source data are provided as a Source Data file.
