# Supplementary material for: The DYT6 dystonia causative protein THAP1 is responsible for proteasome activity via PSMB5 transcriptional regulation
Source: Nat Commun. 2025 Feb 14;16:1600. doi: 10.1038/s41467-025-56867-x (PMC11828994; doi:10.1038/s41467-025-56867-x)
Supplement: Supplementary file 2 — Description of Additional Supplementary Files [file 41467_2025_56867_MOESM2_ESM.docx]

**Description of additional Supplementary Data files**

**File Name:** Supplementary Data 1

**Description**: The results of RNA-seq analysis of HEK293T control and sgTHAP1. Data are presented as mean ± SEM (n = 3 from three biological replicates). Significance was calculated using an unpaired two-tailed Student’s t-test correction. The data relates to Fig.3c.

**File Name:** Supplementary Data 2

**Description:** The results of RNA-seq analysis comparing the E10.5 embryos of mouse control and homozygous C54Y Thap1 mutants. Data represent the mean ± SEM (n = 3 for *Thap1^+/+^* and n = 3 for *Thap1^C54Y/C54Y^* ). Significance was calculated using an unpaired two-tailed Student’s t-test correction. The data relates to Fig.6d.

**File Name:** Supplementary Data 3

**Description:** List of qRT-PCR primers used in this study.
