# Supplementary material for: The DYT6 dystonia causative protein THAP1 is responsible for proteasome activity via PSMB5 transcriptional regulation
Source: Nat Commun. 2025 Feb 14;16:1600. doi: 10.1038/s41467-025-56867-x (PMC11828994; doi:10.1038/s41467-025-56867-x)
Supplement: Supplementary file 6 — Reporting Summary [file 41467_2025_56867_MOESM6_ESM.pdf]

nature portfolio

Corresponding author(s): Shigeo Murata

Last updated by author(s): Jan 14, 2024

Reporting Summary

Nature Portfolio wishes to improve the reproducibility of the work that we publish. This form provides structure for consistency and transparency in reporting. For further information on Nature Portfolio policies, see our Editorial Policies and the Editorial Policy Checklist.

Statistics

For all statistical analyses, confirm that the following items are present in the figure legend, table legend, main text, or Methods section.

n/a	Confirmed	
☐	☒	The exact sample size (*n*) for each experimental group/condition, given as a discrete number and unit of measurement
☐	☒	A statement on whether measurements were taken from distinct samples or whether the same sample was measured repeatedly
☐	☒	The statistical test(s) used AND whether they are one- or two-sided<br>*Only common tests should be described solely by name; describe more complex techniques in the Methods section.*
☒	☐	A description of all covariates tested
☐	☒	A description of any assumptions or corrections, such as tests of normality and adjustment for multiple comparisons
☐	☒	A full description of the statistical parameters including central tendency (e.g. means) or other basic estimates (e.g. regression coefficient) AND variation (e.g. standard deviation) or associated estimates of uncertainty (e.g. confidence intervals)
☐	☒	For null hypothesis testing, the test statistic (e.g. *F*, *t*, *r*) with confidence intervals, effect sizes, degrees of freedom and *P* value noted<br>*Give P values as exact values whenever suitable.*
☒	☐	For Bayesian analysis, information on the choice of priors and Markov chain Monte Carlo settings
☒	☐	For hierarchical and complex designs, identification of the appropriate level for tests and full reporting of outcomes
☒	☐	Estimates of effect sizes (e.g. Cohen's *d*, Pearson's *r*), indicating how they were calculated

Our web collection on statistics for biologists contains articles on many of the points above.

Software and code

Policy information about availability of computer code

Data collection	RT-qPCR data were acquired with Light Cycler 480 Ⅱ (Roche).<br>Western Blots data were acquired with FUSION SL4 400 (Vilber Lourmat).<br>Flow cytometry were performed by Attune NxT Flow Cytometer (Thermo).<br>Peptide activity were measured by Nivo Alpha F HH35000400 (Revvity).<br>The accession number for human ChIP-Atlas ChIP-Seq data (PMID: 30413482) from K562 cells used in the manuscript for THAP1 is GEO: GSM803408.<br>The accession number for the microarray analyses comparing the mouse control and N-CKO for Thap1 (PMID: 28697333) used in the manuscript is GEO: GSE97372.
Data analysis	Graphs and statistical tests were carried out with Prism 9 software (GraphPad Software).. Flow cytometry data were analyzed by Attune NxT software v2.7.0 and FlowJo v10.1.

For manuscripts utilizing custom algorithms or software that are central to the research but not yet described in published literature, software must be made available to editors and reviewers. We strongly encourage code deposition in a community repository (e.g. GitHub). See the Nature Portfolio guidelines for submitting code & software for further information.

Data

Policy information about availability of data

All manuscripts must include a data availability statement. This statement should provide the following information, where applicable:

- Accession codes, unique identifiers, or web links for publicly available datasets
- A description of any restrictions on data availability
- For clinical datasets or third party data, please ensure that the statement adheres to our policy

The results of CRISPR screening sequencing datasets generated and analyzed in this study are not publicly available due to patient privacy and confidentiality but are available from the corresponding author on reasonable request.
The RNA-seq data comparing the HEK293T control and gRNA for THAP1 have been deposited to the DDBJ Sequence Read Archive under the accession code DRR576089-DRR576094. Processed data files have been deposited to the Genomic Expression Archive under the accession code E-GEAD-845.
The accession number for the RNA-seq analysis comparing the E10.5 embryos of mouse control and homozygous C54Y Thap1 mutants, as used in the manuscript, is GSE:283266.
All data supporting the findings of this study are available within the paper. the remaining data are available within the article, supplementary information or Source data file. source data are provide with this paper.All other data supporting the findings of this study are available from the corresponding author on reasonable request.

Research involving human participants, their data, or biological material

Policy information about studies with human participants or human data. See also policy information about sex, gender (identity/presentation), and sexual orientation and race, ethnicity and racism.

Reporting on sex and gender	The findings in our study do not apply to only one gender. Gender was not considered in the study design.
Reporting on race, ethnicity, or other socially relevant groupings	Race/ethnicity was not considered in the study design.
Population characteristics	See above
Recruitment	N/A
Ethics oversight	N/A

Note that full information on the approval of the study protocol must also be provided in the manuscript.

Field-specific reporting

Please select the one below that is the best fit for your research. If you are not sure, read the appropriate sections before making your selection.

☒ Life sciences ☐ Behavioural & social sciences ☐ Ecological, evolutionary & environmental sciences

For a reference copy of the document with all sections, see nature.com/documents/nr-reporting-summary-flat.pdf

Life sciences study design

All studies must disclose on these points even when the disclosure is negative.

Sample size	No statistical method was used to predetermine sample size. Sample sizes were determined basing on previous experiences and similar published studies elsewhere.
Data exclusions	No Data exclusions were applied.
Replication	For all our results at least two experimental replicates were performed.
Randomization	Randomization was not required as no human participants or animal models were reported in this manuscript and experiments were performed on specified cell lines.
Blinding	Experiments were performed blinded when possible. Blinding was not possible for many experiments as especially for cell culture experiments given experimental conditions had to be monitored throughout the experiment. To mitigate this, data were plotted and analyzed at the end of the experiment and measurements and analysis were confirmed independently.

Reporting for specific materials, systems and methods

We require information from authors about some types of materials, experimental systems and methods used in many studies. Here, indicate whether each material, system or method listed is relevant to your study. If you are not sure if a list item applies to your research, read the appropriate section before selecting a response.

Materials & experimental systems

n/a	Involved in the study	
	x	Antibodies
	x	Eukaryotic cell lines
x		Palaeontology and archaeology
	x	Animals and other organisms
x		Clinical data
x		Dual use research of concern
x		Plants

Methods

n/a	Involved in the study	
	x	ChIP-seq
	x	Flow cytometry
x		MRI-based neuroimaging

Antibodies

Antibodies used	Immunoblotting (IB) (Target molecule, target species, host species, clone, dilution, company, catalog number, Lot number)<br>Proteasome 20S α1, α2, α3, α4, α6, α7, β1, β2, β3, β4, β5, β6, and β7subunit, Mouse, Rabbit, N/A, 1:1000, Homemade, N/A<br>Proteasome 20S α5 subunit, Mouse, Mouse, N/A, 1:1000, Homemade, N/A<br>Proteasome 19S Rpt1, Rpt2, Rpt4, and Rpt5 subunit, Human, Rabbit, N/A, 1:1000, Homemade, N/A<br>Proteasome 19S Rpt3, and Rpt6 subunit, Human, Mouse, N/A, 1:1000, Homemade, N/A<br>Proteasome 19S Rpn1, Rpn3, Rpn5, Rpn6, Rpn7, Rpn8, Rpn9, Rpn10 and Rpn12subunit, Human/Mouse, Rabbit, N/A, 1:1000, Homemade, N/A<br>Proteasome 19S Rpn2 subunit, Human/Mouse, Mouse, N/A, 1:1000, Homemade, N/A<br>Proteasome assembly chaperone PAC2, and Ump1, Human/Mouse, Rabbit, N/A, 1:1000, Homemade, N/A<br>Ubiquitin, Human, Mouse, FK2, 1:1000, Nippon Bio-Test Laboratories, NBT-MFK003<br>β-Actin, Human/Mouse, Mouse, 6D1, 1:1000, MBL, M177-3<br>THAP1, Human/Mouse/Rat, Rabbit, N/A, 1:1000, Proteintech, 12584-1-AP<br>FLAG, Human/Mouse, Mouse, N/A, 1:1000, Sigma Millipore, F1804<br>HRP-conjugated rabbit anti-mouse Ig, 1:20000, Jackson ImmunoResearch Laboratories, 315-035-048<br>HRP-conjugated goat anti-rabbit Ig, 1:20000, Jackson ImmunoResearch Laboratories, 111-035-144<br>The antibodies for α3, α6, β1, β5, β7, Rpt3, Rpt6, and Rpn8 were described previously (Hamazaki et al., 2005; Hamazaki et al., 2007; Hirano et al., 2007; Bai et al., 2014; Hamazaki et al., 2015).
Validation	All commercial antibodies were validated by the manufactures and were previously used in peer reviewed works. Antibodies against α3, α6, β1, β5, β7, Rpt3, Rpt6, and Rpn8 were previously used for publication Hamazaki et al., 2005; Hamazaki et al., 2007; Hirano et al., 2007; Bai et al., 2014; Hamazaki et al., 2015.

Eukaryotic cell lines

Policy information about cell lines and Sex and Gender in Research

Cell line source(s)	HEK293T (ATCC Cat. CRL-3216) , U2OS (ATCC Cat. HTB-96) , HeLa (ATCC Cat. CCL-2) and SH-SY5Y(ATCC Cat. CRL-2266) cells were purchase from American Type Culture Collection (ATCC) .
Authentication	HEK293FT cells were acquired from Thermo Fisher
Mycoplasma contamination	all cell lines tested negative for mycoplasma by DAPI staining
Commonly misidentified lines<br>(See ICLAC register)	no commonly misidentified lines were used

Animals and other research organisms

Policy information about studies involving animals; ARRIVE guidelines recommended for reporting animal research, and Sex and Gender in Research

Laboratory animals	All mice were C57BL/6 background and used at 8-16 weeks old. Both male and females were used in the study. Thap1 C54Y/+ mutant mice were generated with CRISPR/Cas9 methods. All mice were housed in a 12h/12h light/dark cycles at ambient temperature of 20-24 ° C and a humidity range 40-60%. These were also incorporated in the manuscript.
Wild animals	The study did not involve wild animals.
Reporting on sex	Both male and females were used in the study. The study did not involve sexual.
Field-collected samples	No field samples were collected.
Ethics oversight	All animal experiments were performed according to the protocol that approved by the University of Tokyo.

Note that full information on the approval of the study protocol must also be provided in the manuscript.

Plants

Seed stocks	N/A
Novel plant genotypes	N/A
Authentication	N/A

ChIP-seq

Data deposition

- [x] Confirm that both raw and final processed data have been deposited in a public database such as GEO.
- [x] Confirm that you have deposited or provided access to graph files (e.g. BED files) for the called peaks.

Data access links<br>*May remain private before publication.*	ChIP Seq analyses are conducted for public data previously published in the original study. The accession number for human ChIP-Atlas ChIP-Seq data from K562 cells used in the manuscript for THAP1 is GEO: GSM803408 and SRX100453 of ChIP-Atlas.
Files in database submission	N/A
Genome browser session<br>(e.g. UCSC)	N/A

Methodology

Replicates	N/A
Sequencing depth	N/A
Antibodies	N/A
Peak calling parameters	N/A
Data quality	N/A
Software	The visualization software of public ChIP-seq data is IGV.

Flow Cytometry

Plots

Confirm that:

- [x] The axis labels state the marker and fluorochrome used (e.g. CD4-FITC).
- [x] The axis scales are clearly visible. Include numbers along axes only for bottom left plot of group (a 'group' is an analysis of identical markers).
- [x] All plots are contour plots with outliers or pseudocolor plots.
- [x] A numerical value for number of cells or percentage (with statistics) is provided.

Methodology

Sample preparation	Described in the paper
Instrument	Attune NxT Flow Cytometer (Thermo Fisher)
Software	Attune NxT software v2.7.0, FlowJo v10.1
Cell population abundance	For all studies about 20,000 cells were collected per sample.
Gating strategy	For mCherry-P2A-ZsGreen-mODC proteasome activity assay, first, doublets were excluded with a FSC-W (y) vs FSC-H (x) dot

plot. "Single Cells" were analyzed for the detection of the flourescence of mCherry and ZsGreen for proteasome activity in dot plot .

☒ Tick this box to confirm that a figure exemplifying the gating strategy is provided in the Supplementary Information.
